# The nature of hydrogen-bonding interaction in the prototypic hybrid halide perovskite, tetragonal CH_3_NH_3_PbI_3_

**DOI:** 10.1038/srep21687

**Published:** 2016-02-19

**Authors:** June Ho Lee, Jung-Hoon Lee, Eui-Hyun Kong, Hyun Myung Jang

**Affiliations:** 1 Department of Materials Science and Engineering, and Division of Advanced Materials Science (AMS), Pohang University of Science and Technology (POSTECH), Pohang 790-784, Republic of Korea; 2 Korea Atomic Energy Research Institute (KAERI), Yuseong-Gu, Daejeon 305-353, Republic of Korea

## Abstract

In spite of the key role of hydrogen bonding in the structural stabilization of the prototypic hybrid halide perovskite, CH_3_NH_3_PbI_3_ (MAPbI_3_), little progress has been made in our in-depth understanding of the hydrogen-bonding interaction between the MA^+^-ion and the iodide ions in the PbI_6_-octahedron network. Herein, we show that there exist two distinct types of the hydrogen-bonding interaction, naming *α*- and *β*-modes, in the tetragonal MAPbI_3_ on the basis of symmetry argument and density-functional theory calculations. The computed Kohn-Sham (K-S) energy difference between these two interaction modes is 45.14 meV per MA-site with the *α*-interaction mode being responsible for the stable hydrogen-bonding network. The computed bandgap (*E*_*g*_) is also affected by the hydrogen-bonding mode, with *E*_*g*_ of the *α*-interaction mode (1.73 eV) being significantly narrower than that of the *β*-interaction mode (2.03 eV). We have further estimated the individual bonding strength for the ten relevant hydrogen bonds having a bond critical point.

Organic-inorganic hybrid perovskite-based solar cells have revolutionized the photovoltaic landscape^1^,^2^,^3^,^4^,^5^,^6^,^7^,^8^,^9^ as they have demonstrated unprecedentedly high power conversion efficiencies (PCEs), combined with low cost. Their electrical PCE increases extremely rapidly and has reached ~19% in 2013, up from ~3% in 2009^1^,^2^,^3^,^4^,^5^,^6^,^7^,^8^,^9^. The observed unusually high PCEs are currently attributed to several relevant physical factors that include low optical bandgaps^10^, large absorption coefficients^1^, and long carrier diffusion lengths^11^,^12^. In addition to high PCEs, hybrid halide perovskites of RMX_3_-type show a remarkable capability of demonstrating diverse photovoltaic properties by suitable substitution or modification of organic molecules (R)^13^ or metal (M) ions^14^ in the hybrid perovskite lattice.

Among numerous hybrid perovskites, a group of halides having the stoichiometry of CH_3_NH_3_PbX_3_ (abbreviated as MAPbX_3_, where X = Cl, Br, I) is the most widely studied and considered to be a typical hybrid perovskite. It is known that MAPbX_3_ undergoes consecutive phase transitions with the following sequence: cubic-tetragonal-orthorhombic allotropic phases with decreasing temperature^15^,^16^. In case of the low-temperature orthorhombic phase, the organic molecules (MAs) are well oriented to maximize the hydrogen-bonding interaction between the MA groups and the corner-shared PbI_6_ octahedra^17^. Thus, the orientation of the MA group and consequently the positions of hydrogen atoms are well defined in the orthorhombic phase. In the room-temperature-stable tetragonal phase or in the high-temperature cubic phase, on the contrary, the organic MA molecules are randomly oriented with no clear orientational correlation between them^18^. The configuration of the resulting hydrogen-bonding network is thus extremely complicated, which would lead to numerous local minima in the potential-energy surfaces. Thus, in the case of tetragonal or cubic phase, it seems to be extremely difficult to find the optimum configuration which corresponds to the global minimum in the energy-configuration space of MAPbX_3_.

According to the previous theoretical study, the organic cations of different sizes and hydrogen-bonding interactions [*e.g*., CH_3_NH_3_^+^ and (NH_2_)_2_CH^+^] are capable of affecting the optical bandgaps of RPbI_3_-based perovskites^19^. Similarly, Filip *et al.*^20^ have experimentally shown that tunable optical bandgaps are achieved by controlling the degree of the PbI_6_ octahedral tilting through the steric size of the molecular cation. According to these two studies^19^,^20^, the optical bandgap can be reduced by decreasing the degree of the octahedral tilting, which, in turn, can be achieved by adjusting the degree of the hydrogen-bonding interaction between the halides and H atoms bonded to the MA group. Several other studies^21^,^22^,^23^,^24^,^25^ also indicate the important role of the MA^+^-ion orientation and, thus, the hydrogen-bonding interaction in controlling the core properties of the MAPbX_3_-based perovskite solar cells, which includes the enhanced carrier diffusion length^21^, the ferroelectric photovoltaic effect^22^, and the interplay of the MA-dipole orientation with the stability of perovskite structure^24^,^25^.

In spite of the key role of the MA-dipole orientation and consequent hydrogen-bonding interaction, little progress has been made in our systematic understanding of (i) the stable configuration of MA^+^-ions in the perovskite unit cell and (ii) the nature and strength of the hydrogen bonding between the MA^+^-ion and the halide (X) ions in the PbX_6_-octahedron network. Herein, we show that there exist two distinct types of the hydrogen-bonding interaction in the tetragonal phase which is relevant to room-temperature performance of the prototypic MAPbI_3_-based solar cells. On the basis of symmetry consideration of the PbI_6_-octahedron network, we will predict the possibility of existence of two distinct chemical environments for the MA^+^-ion orientation in the tetragonal phase and computationally show that one of these two is responsible for the stable hydrogen-bonding interaction between the MA^+^-ion and the surrounding PbI_6_-octahedron cages.

## Results and Discussion

### Two Distinct Environments for the Organic-cation Orientation

**Figure 1:**
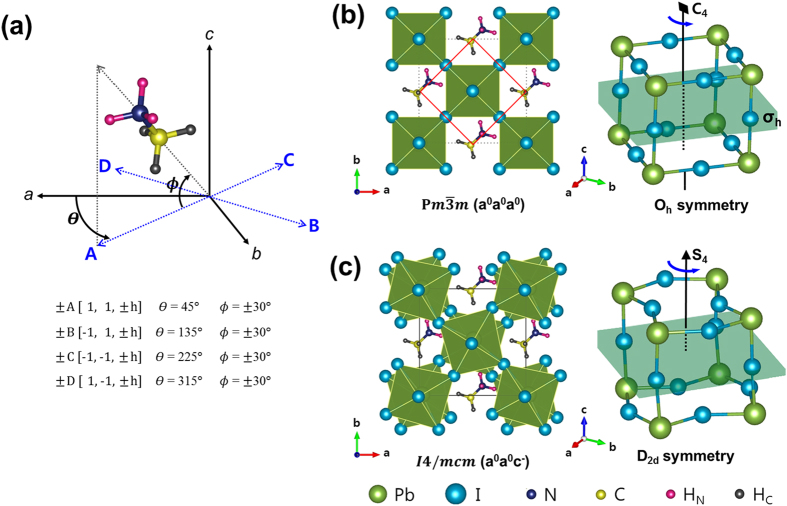
**(a)**Eight preferred orientations of the organic MA^+^-ion (i.e., C-N bond axis) within the perovskite cavity. Herein, A, B, C, and D represent the projection of the MA^+^-ion orientations on the *a-b* plane, as measured by the azimuthal angle *θ*. The orientation of the MA^+^-ion with respect to the *a-b* in-plane is represented by the tilting angle *ϕ*. According to Quarti *et al.*^24^ the two optimum *ϕ* angles are ±30°. However, we have found that the optimum *ϕ* angle depends sensitively on the hydrogen-bonding interaction mode (See the text for details). **(b)** Crystal structure of the high-temperature cubic 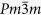 phase viewed from the *c*-axis (left-hand side). The corresponding Pb-I inorganic cage characterized by the C_4_-rotation axis and the mirror plane 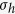 perpendicular to the C_4_ axis (right-hand side). **(c)** Crystal structure of the tetragonal *I4/mcm* phase viewed from the *c*-axis (left-hand side). The corresponding Pb-I inorganic cage characterized by the improper S_4_-rotation axis (right-hand side).

Quarti *et al.*^24^ computationally showed that a set of polar (ferroelectric-like) structures formed by a preferred MA^+^-ion orientation is more stable, in general, than a set of apolar (antiferroelectric-like) structures formed by an isotropic distribution of the MA dipoles, which indicates an important role of the MA^+^-ion orientation in the stability of the perovskite lattice. Molecular dynamics computations^26^ and first-principles study^27^ further showed that for both cubic and tetragonal phases, the MA^+^ cations are oriented parallel to the facial direction of the inorganic cage. On the basis of these theoretical studies, one can describe the orientation of the organic cations (MA^+^) in the tetragonal phase by 8 different initial orientations^24^. These eight orientations are described by two characteristic angles, *θ* and *ϕ*, and are graphically illustrated in Fig. 1a, where *a*, *b*, and *c* denote the three crystallographic axes of the tetragonal perovskite lattice. In the figure, *θ* defines the angle between the *a*-axis (i.e., *x*-direction) and the projection of the MA^+^-ion orientation within the *ab*-plane. Thus, the four possible projection vectors are oriented along 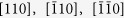, and 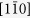, which respectively correspond to the MA cations lying within the *ab*-plane with the *θ* angles of 45° (for A), 135° (for B), 225° (for C), and 315° (for D). With respect to the *ab*-plane, the MA cations have two symmetric preferred orientations, *ϕ* = ±30° 24, where *ϕ* is the tilting angle of the C-N bond axis with respect to the *ab*-in-plane (Fig. 1a).

Figure 1b shows the crystal structure of the high-temperature cubic phase composed of the central PbI_6_-octahedron cage and the surrounding MA^+^ ions. In the cubic phase which is represented by the 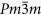 space-group symmetry, the corner-shared PbI_6_ octahedral frame does not show any tendency of the octahedral tilting along all three directions, *a*, *b*, and *c*. Thus, the cubic phase is represented by 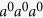 in the Glazer’s notation. Figure 1c shows the tilted three-dimensional structure of the room-temperature-stable tetragonal phase which belongs to the *I4/mcm* space group. In this tetragonal structure, the PbI_6_ octahedra do not show any alternative tilting along the *a-* and *b*-axes but exhibit out-of-phase tilting along the *c*-axis, which is in accordance with the 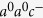 tilt pattern in the Glazer’s notation.

Let us now consider the difference in the point-group symmetry of the PbI_6_-octahedron cage between these two relevant phases. In the cubic phase, the PbI_6_ octahedral network belongs to *O*_*h*_ point group which is characterized by the principal 4-fold rotation axis along the *c*-axis (*C*_*4*_) and the mirror plane perpendicular to this *C*_*4*_ axis 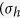; Fig. 1b). Owing to the *C*_*4*_ symmetry, a set of the following four distinct orientations of the C-N bond axis is under the same chemical environment: {+*A*, +*B*, +*C*, +*D*}. Similarly, a set of the orientations, {−*A*, −*B*, −*C*, −*D*}, at a given MA-site is chemically equivalent in the cubic phase. Owing to the 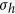 symmetry, however, the two orientations having the same *θ* angle but with two opposite *ϕ* values (*e.g.*, +*A* and –*A* orientations; Fig. 1a) are under the same chemical environment. Thus, in the cubic phase, the PbI_6_-octahedron cage provides all eight possible orientations of MA, {±A, ±B, ±C, ±D}, with the same chemical environment. This symmetry argument is graphically illustrated in Figure S1 of the Supplementary Information.

**Figure 2:**
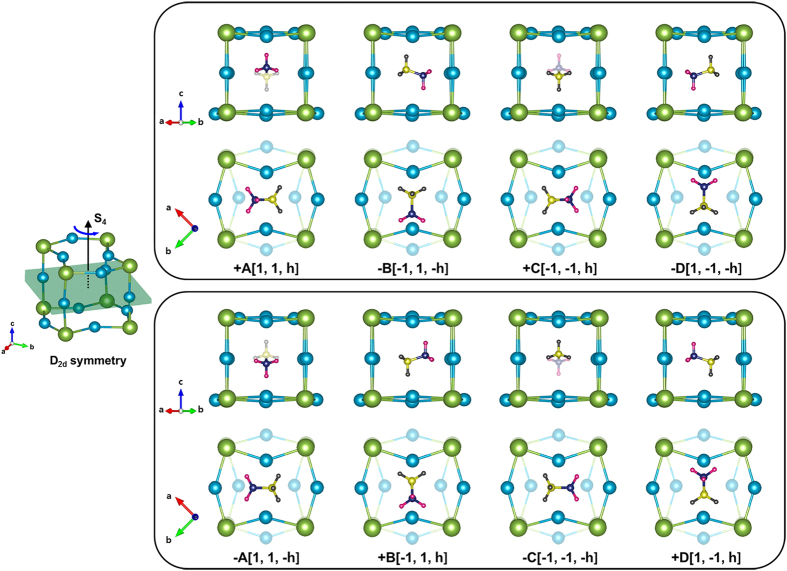
Graphical illustration of the two distinct sets of the MA^+^-ion orientations (at a given MA-site) in the tetragonal MAPbI_3_ with D_*2d*_ symmetry. **(upper panel)** The central MA^+^-ion viewed along [110] (upper row) and viewed along [001] (lower row) for a set of the four distinct orientations, {+*A*, −*B*, +*C*, −*D*}. **(lower panel)** The central MA^+^-ion viewed along [110] (upper row) and viewed along [001] (lower row) for a set of the four distinct orientations, {−*A*, +*B*, −*C*, +*D*}.

In the room-temperature-stable tetragonal phase, on the contrary, the PbI_6_ octahedral network belongs to *D*_*2d*_ point group owing to the 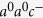 tilt pattern. Thus, the PbI_6_-cage network is characterized by the S_4_ improper rotation axis along the *c*-axis (Fig. 1c). Because of the S_4_ improper rotation, a set of the following four distinct orientations of the C-N bond axis (at a given arbitrary MA-site) is under the same chemical environment: {+*A*, −*B*, +*C*, −*D*}. Similarly, a set of the orientations, {−*A*, +*B*, −*C*, +*D*}, at a given MA-site is chemically equivalent in the tetragonal phase. Consequently, there exist two distinct chemical environments (also, energetically non-degenerate) for the MA^+^-ion orientation in the tetragonal phase. These two distinct sets of orientations are graphically illustrated in Fig. 2: {+*A*, −*B*, +*C*, −*D*} in the upper panel and {−*A*, +*B*, −*C*, +*D*} in the lower panel.

**Figure 3:**
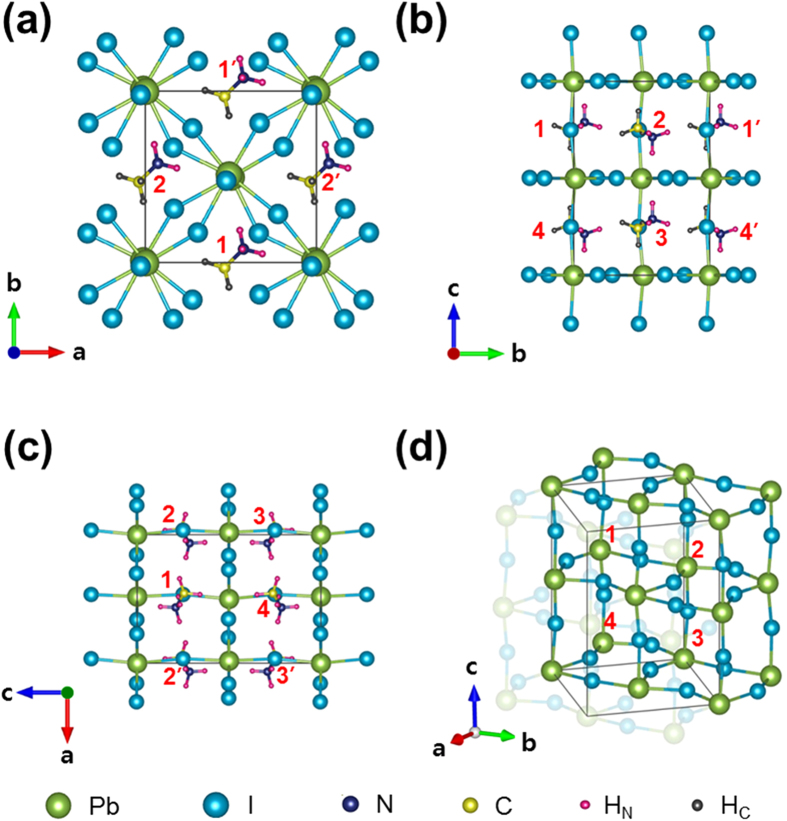
The unit-cell structure of the tetragonal MAPbI_3_ with the marked four distinct MA-sites. **(a)** The unit-cell structure viewed from the *c*-axis. Herein, the four MA dipoles (1, 2, 1′ and 2′) lie on the same *a-b* plane. **(b)** The unit-cell structure viewed from the *a*-axis. **(c)** The unit-cell structure viewed from the *b*-axis. **(d)** The structure of tetragonal MAPbI_3_ unit cell viewed from an arbitrary axis.

The unit-cell structure of MAPbI_3_ with the marked four distinct MA-sites is depicted in Fig. 3. As displayed in Fig. 3a, the four MA dipoles (1, 2, 1′, and 2′) are located at the same *a-b* plane. When the cell is viewed from the *a*-axis (Fig. 3b), the 1^st^ and 2^nd^ MA-sites are on the same *a-b* plane but the 3^rd^ and 4^th^ sites are located at a different *a-b* plane which is (*c*/2) away from the former *a-b* plane along the *c*-axis of the tetragonal *I4/mcm* cell. Thus, the distance between the 1^st^ and 2^nd^ sites (or equivalently, between the 3^rd^ and 4^th^ sites) is given by 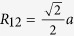, where *a* is the *a*-axis lattice parameter.

### Two Distinct Modes of Hydrogen-bonding Interaction

We have examined the above made proposition on the existence of two non-equivalent chemical environments by investigating the MA-ion orientation in the tetragonal phase on the basis of *ab initio* density-functional theory (DFT) calculations. We used the experimental lattice parameters 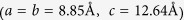
16 as the input parameters of our DFT calculations and subsequently obtained the optimized local structures of MAPbI_3_ by applying the structure relaxation (i.e., relaxation of the internal positions at a fixed unit-cell volume). However, the volume relaxation method also gives essentially the same DFT optimized results that include the Kohn-Sham (K-S) energy and the equilibrium tilting angle, *ϕ*.

We have chosen two orientations, +A and –A, to examine the existence of two non-degenerate chemical environments at a particularly chosen MA site. However, our discussion is also valid for other pairs of the MA orientations (*e.g*., +C & –C). The DFT optimized value of *θ* is 45° for both +A and –A orientations [Fig. 1a]^24^. However, the optimum tilting angle (*ϕ*) which corresponds to the minimum in the K-S energy depends sensitively on the MA^+^-ion orientation: ~+22° for +A orientation and ~+5° for –A initial orientation. It is interesting to notice that the optimum relaxed tilting angle (*ϕ*) for the –A initial orientation is ~+5°, instead of yielding a negative value. This is quite surprising since the input *ϕ* value for the –A orientation (usually 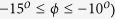 corresponds to a set of the degenerate orientations, {−*A*, +*B*, −*C*, +*D*} but the relaxed equilibrium *ϕ* value then belongs to a set of the opposite orientations, {+*A*, −*B*, +*C*, −*D*}.

It can be shown that for the –A initial orientation, the inconsistency between the symmetry prediction and the DFT optimized result stems mainly from the hydrogen-bonding interaction between the MA^+^-ion and I^**−**^ ions in the PbI_6_-octahedron network. More specifically, the DFT optimized result fully reflects the site-specific hydrogen-bonding effect. On the contrary, the symmetry prediction is purely based on the *D*_*2d*_ structural symmetry of the PbI_6_-octahedron network without considering this site-specific hydrogen-bonding interaction between the MA^+^-ion and I^**−**^ ions. Because of this simplification, the symmetry prediction can only be used as an initial guideline. In actual DFT calculations, we have adopted the structure relaxation at a fixed unit-cell volume, instead of using the volume relaxation, by considering computational efficiency and cost. As mentioned previously, however, the structure relaxation gives essentially the same DFT optimized results as the volume relaxation method.

The above described extraordinary result indicates that the particular MA-site chosen in the present DFT calculations strongly prefers the +A orientation to the –A orientation. Let us call this particular site as the 1^st^ MA-site, as shown in Fig. 3. Indeed, the calculated K-S energy difference between the two orientations is as large as 45.14 meV per MA-site. Thus, a set of the orientations, {−*A*, +*B*, −*C*, +*D*}, does not practically exist at the 1^st^ MA-site though the symmetry argument predicts its existence. Consequently, we end up with a positive equilibrium *ϕ* even if we use a negative input *ϕ* value for the –A initial orientation. In our calculations of the K-S energy for the +A orientation at the 1^st^ MA-site, we have chosen the site-dependent dipole configuration of [+A,-A,+A,-A] which denotes the MA^+^-ion orientations of +A, -A, +A, and –A at 1^st^, 2^nd^, 3^rd^, and 4^th^ sites, respectively. It can be shown that this particular MA^+^-ion configuration corresponds to the symmetry-allowed lowest energy configuration (See Subsection “Remarkably Simplified Dipole Configurations by Considering Structural Symmetry.”). On the contrary, the [-A,+A,-A,+A] initial configuration was used to evaluate the K-S energy for the −A orientation at the same 1^st^ MA-site. Thus, the K-S energy difference between these two distinct dipole configurations, [+A,-A,+A,-A] and [-A,+A,-A,+A], is as high as 180.56 meV (=45.14 × 4).

**Figure 4:**
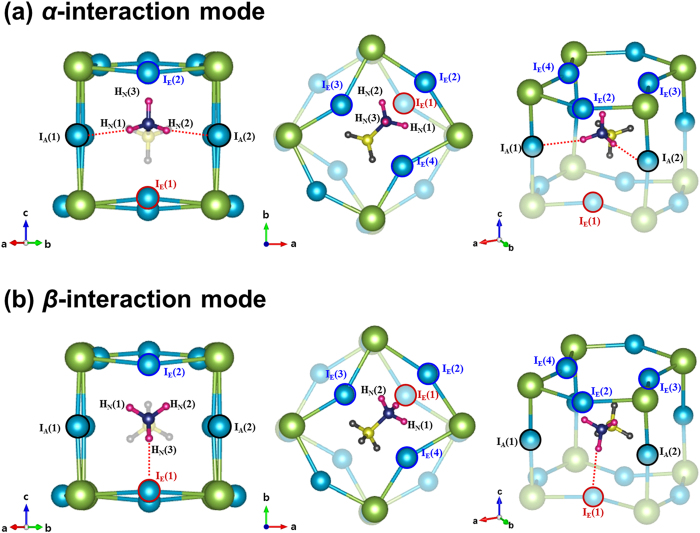
Illustration of the two distinct modes of the hydrogen-bonding interaction between the MA^+^-ion and the surrounding PbI_6_-octahedron cages. **(a)** *α*-interaction mode viewed along [110] (left), viewed along [001] (center), and viewed from an arbitrary axis (right). **(b)** *β*-interaction mode viewed along [110] (left), viewed along [001] (center), and viewed from an arbitrary axis (right).

Let us define the hydrogen-bonding interaction mode that corresponds to the tilting angle (*ϕ*) of +22° as the *α*-interaction mode. Similarly, let us denote the hydrogen-bonding interaction mode corresponding to the tilting angle (*ϕ*) of +5° as the *β*-interaction mode. Recall that the input *ϕ* value for the *β*-interaction mode is negative although the relaxed value is positive, ~+5°. As mentioned previously, the K-S energy difference between these two tilting-angle states is 45.14 meV per MA-site (i.e., per formula unit). The *α*-interaction mode with *ϕ* value of ~+22° is structurally illustrated in Fig. 4a by showing the 1^st^ MA-site (at center) and the surrounding PbI_6_-octahedron cages. On the other hand, the *β*-interaction mode with *ϕ* value of ~+5° is structurally depicted in Fig. 4b. Herein, the apical (axial) iodine atoms in the PbI_6_-octahedron cage are denoted by I_A_, whereas the equatorial iodine atoms are marked with I_E_. The three hydrogen atoms bonded to the nitrogen (N) atom are denoted by H_N_ while the three hydrogen atoms connected to the carbon (C) atom are designated by H_C_.

Among the three H_N_ atoms that are directly involved in hydrogen bonds, H_N_(3) atom shows the most prominent difference in the hydrogen-bonding interaction between the *α*- and *β*-modes. In principle, H_N_(3) is capable of simultaneously interacting with three different equatorial iodine atoms, I_E_(2), I_E_(3), and I_E_(4), in the *α*-interaction mode. On the contrary, H_N_(3) can interact only with I_E_(1) in the *β*-interaction mode (See Figure S2 of the Supplementary Information). According to the computed bond length and energy (Table 1), three hydrogen bonds are by far outstanding among the 21 possible H-I interactions (11 for the *α*-mode and 10 for the *β*-mode) having a bond critical point where the gradient of the local electron density, 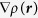, is zero. These are: H_N_(1)∙∙∙I_A_(1) and H_N_(2)∙∙∙I_A_(2) in the *α*-interaction mode and H_N_(3)∙∙∙I_E_(1) in the *β*-interaction mode (denoted by dotted red lines in Fig. 4). These three hydrogen bonds are named ‘the dominant hydrogen bonds.’

### Bonding-mode-dependent Band Structure

**Figure 5:**
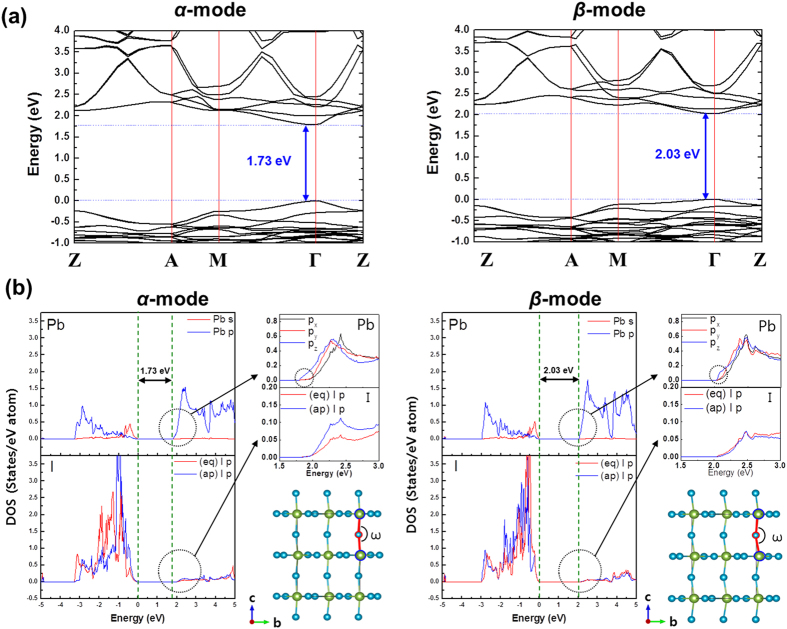
The band structure and the partial density of states (PDOS) of the tetragonal MAPbI_3_ cell for the two distinct modes of the hydrogen-bonding interaction. **(a)** The band structure of the tetragonal MAPbI_3_ cell under the *α*-interaction mode (left) *versus* the band structure under the *β*-interaction mode (right). The *ab initio* band-structure calculations were performed along high-symmetry surface ***k***-vectors of the first Brillouin zone. **(b)** The computed PDOS of the tetragonal MAPbI_3_ cell under the *α*-interaction mode (left) *versus* the PDOS under the *β*-interaction mode (right). The Pb *6p*_*z*_-I 5p* orbital overlapping at the CBM is reasoned to be closely correlated with the bandgap reduction under the *α*-interaction mode.

We have examined the effect of the hydrogen-bonding mode on the band structure of the tetragonal MAPbI_3_ cell. The computed band structures are similar to those previously reported by Mosconi *et al.*^23^. However, as indicated in Fig. 5a, the bandgap (*E*_*g*_) at the zone-center Γ-point is significantly affected by the hydrogen-bonding mode. We have further examined the partial density-of-states (PDOS) to resolve the atomic-scale origin of this bonding-mode-dependent bandgap. As indicated in Fig. 5b, the conduction-band minimum (CBM) is characterized by the Pb *6p* orbitals, which is irrespective of the hydrogen-bonding interaction mode. On other hand, the valence-band maximum (VBM) is featured by the Pb *6s* and I *5p* orbitals. A detailed analysis of the wavefunction-character indicates that the Pb *6p*-I 5p* anti-bonding orbital corresponds to the overlapping at the CBM while the Pb *6s*-I 5p* anti-bonding orbital represents the VBM. It is interesting to notice that in the case of the *α*-interaction mode, the PDOS of the Pb *6p*_*z*_ orbital at the CBM further penetrates into a lower energy region (down to 1.73 eV above the VBM; Fig. 5b). This lowers the CBM value with respect to the VBM, leading to the bandgap reduction in the case of the *α*-interaction mode.

In addition, the Pb *6p*_*z*_ orbital is expected to show a certain degree of the orbital overlapping with the apical I 5p* orbital under the *α*-interaction mode. A careful examination of the PDOS indeed shows that the PDOS for the apical I 5p* (ap) orbital is slightly higher than that for the equatorial I 5p* (eq) orbital near the CBM under the *α*-interaction mode (Fig. 5b). Owing to the slightly enhanced Pb *6p*_*z*_-I 5p* orbital overlapping at the CBM, it is predicted that the angle between Pb-(*ap*)I-Pb under the *α*-interaction mode is closer to 180° than the corresponding angle under the *β*-interaction mode. Indeed, our *ab initio* DFT calculations showed that the Pb-(*ap*)I-Pb angle under the *α*-interaction mode (*ω* = 168.6°) is substantially closer to 180° than the Pb-(*ap*)I-Pb angle under the *β*-interaction mode (*ω* = 160.3°).

### Characteristic-angle-dependent Kohn-Sham Energy

**Figure 6:**
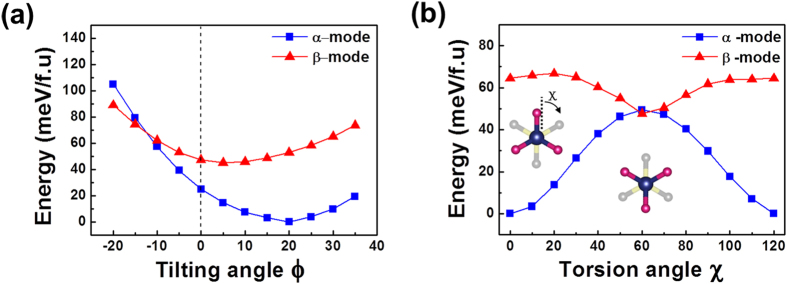
The computed Kohn-Sham energy plotted as a function of **(a)** the tilting angle *ϕ* and **(b)** the torsion angle χ, clearly showing the effect of the hydrogen-bonding interaction mode on the two equilibrium angles. Notice that the initially set orientation of the MA^+^-ion is +A (i.e., positive *ϕ*) for the *α*-interaction mode and –A (i.e., negative *ϕ*) for the *β*-interaction mode.

We have then examined the orientation-dependent K-S energy to assess whether the DFT optimized *α*-interaction mode corresponds to the most stable configuration (in a single unit cell) or not. The orientation of the C-N bond axis is determined by the following three characteristic angles: *θ* (azimuthal angle), *ϕ* (tilting angle), and *χ* (torsion angle), where *χ* defines the rotation angle of the C-N bond axis^17^. For a fixed MA^+^-ion orientation, both *α*- and *β*-interaction modes have a common azimuthal *θ*-angle. For instance, *θ* = 45° for ±A-orientations (Fig. 1a). Thus, we have examined *ϕ*- or *χ*-dependent K-S energy. In Fig. 6a, the computed K-S energy is plotted as a function of the tilting angle, *ϕ*, which indicates the equilibrium *ϕ*-angle for the 1^st^ (or 3^rd^) site is +22° and +5° for *α*- and *β*-interaction modes, respectively. In case of the torsion angle, the K-S energy for the *β*-interaction mode shows its maximum when *χ* is at 0° or 120° while the K-S energy shows its minimum when *χ* is at 60° (Fig. 6b). Contrary to this, the K-S energy shows a reverse trend for the *α*-interaction mode. In this case, a pronounced increase in the K-S energy occurs upon increase in the torsion angle (*χ*) from 0° to 60° or upon decrease in *χ* from 120° to 60° (Fig. 6b). This increase in the K-S energy can be understood in terms of the rupture of the relevant hydrogen bonds upon the torsion of the C-N bond axis from its equilibrium *χ* values, 0°, 120°, *etc* (Fig. 4a).

For each interaction mode, the orientation-dependent energy is described by three characteristic variables, *θ*, *ϕ*, and *χ*. Under the thermodynamic equilibrium, the K-S energy should be its true minimum, simultaneously satisfying the two criteria: 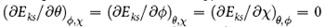
and 
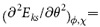
 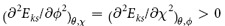 in the three-dimensional 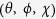
–space. Thus, for the *α*-interaction mode, the equilibrium *ϕ* and *χ* angles deduced from Fig. 6 correspond to the most stable state in a single unit cell.

According to the computed K-S energy shown in Fig. 6b, the activation free-energy for the C-N bond rotation is 49.4 meV for the *α*-interaction mode while it is 16.9 meV for the *β*-interaction mode. This suggests that the net hydrogen-bonding strength in the *α*-interaction mode is much stronger than that in the *β*-interaction mode. We will quantitatively examine this important point in Subsection “Evaluation of Individual Hydrogen-bonding Strength”. Since the room-temperature thermal energy is 25.7 meV, an effectively free torsional motion of the C-N bond axis is expected in the *β*-interaction mode but not in the *α*-interaction mode. According to the transition-state theory^28^, the frequency of the free torsional rotation is estimated to be: 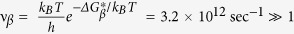 for the *β*-interaction mode at 300 K.

We are in a position to summarize the main difference in the organic MA^+^-ion orientation between the *α*- and *β*-interaction modes: (i) The equilibrium *ϕ*-angle is +22° in the *α*-interaction mode while it is +5° in the *β*-interaction mode. (ii) The orientation relationship of the −NH_3_ group in the *β*-interaction mode with χ = 60° (Fig. 6b) can be reproduced by the 180° rotation of the N-H_N_(3) bond axis of the −NH_3_ group in the *α*-interaction mode (χ = 0° or 120°) along the *c*-axis. This can be identified by examining the two left-hand side illustrations of Fig. 4. On the other hand, both *α*- and *β*-interaction modes have a common *θ*-angle, as mentioned previously.

### Remarkably Simplified Dipole Configurations by Considering Structural Symmetry

Let us begin our discussion by examining conceivable MA-dipole orientations that satisfy the symmetry rule for a given MA-site in the perovskite cell. For this, we have particularly chosen the 1^st^ MA-site among four possible sites in a given perovskite cell (Fig. 3). By considering the restriction imposed by the structural symmetry, we have shown that a set of the dipole orientations, {+*A*, −*B*, +*C*, −*D*}, is allowed at the 1^st^ MA-site. On the other hand, a set of the orientations, {−*A*, +*B*, −*C*, +*D*}, is practically prohibited at the 1^st^ MA-site (Subsection “Two Distinct Modes of Hydrogen-bonding Interaction”). According to the DFT calculations, the +A orientation of the MA^+^-ion at the 1^st^ MA-site is much more stable than the –A orientation (Subsection “Two Distinct Modes of Hydrogen-bonding Interaction”). One can directly apply this symmetry rule of {+*A*, −*B*, +*C*, −*D*} to the ±C orientation. On the contrary, the reverse is true for ±B and ±D orientations. Specifically, the –B (or –D) orientation is much more stable than the +B (or +D) orientation at the 1^st^ MA-site.

**Figure 7:**
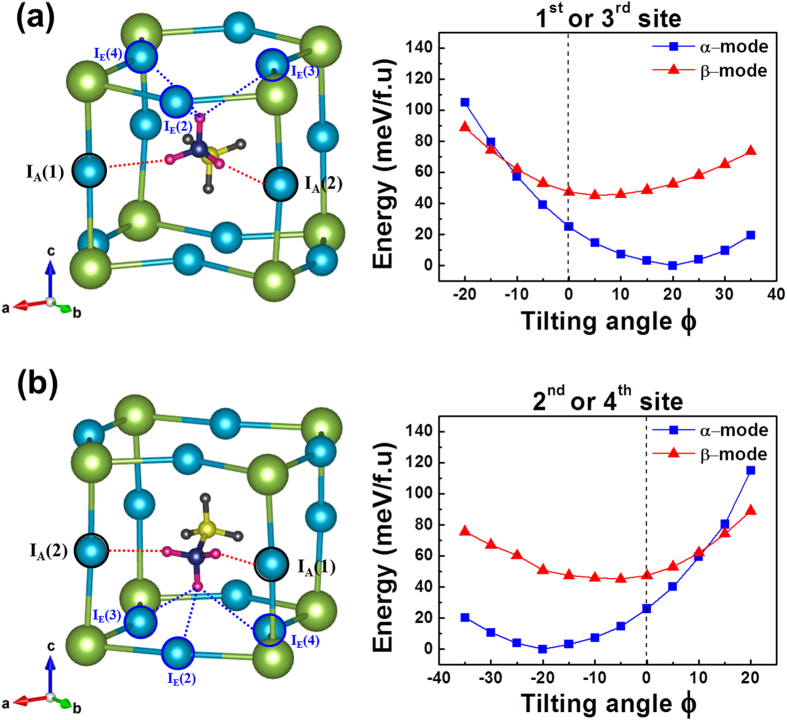
**(a)**The central MA^+^-ion with the +A (or +C) orientation (left). The +A (or +C) orientation with a positive equilibrium tilting angle does correspond to the stable MA dipole at the 1^st^ or 3^rd^ MA-site in the tetragonal MAPbI_3_ cell (right). **(b)** The central MA^+^-ion with the −A (or −C) orientation (left). The −A (or −C) orientation with a negative equilibrium tilting angle does correspond to the stable MA dipole at the 2^nd^ or 4^th^ MA-site (right). Contrary to the above case, the –B (or –D) orientation represents the stable dipole at the 1^st^ or 3^rd^ MA-site while +B (or +D) orientation corresponds to the stable dipole at the 2^nd^ or 4^th^ MA-site of the tetragonal MAPbI_3_ cell.

Let us now extend the above argument to the remaining three MA-sites in the tetragonal unit cell (Fig. 3). On the basis of the translational symmetry of the tetragonal MAPbI_3_ cell, the above symmetry rule can be directly applied to the 3^rd^ site. In other words, the +A (+C) orientation is much more stable than the –A (–C) orientation at the 3^rd^ site, regardless of the hydrogen-bonding interaction mode. Thus, the calculated *ϕ*-dependent K-S energy for the 1^st^ MA-site (Fig. 6a) can be extended to the 3^rd^ MA-site, as shown in Fig. 7a. On the contrary, the reverse is true for the 2^nd^ and 4^th^ sites: the –A (or –C) orientation with a negative tilting angle *ϕ* is much more stable than the +A (or +C) orientation at the 2^nd^ or 4^th^ site, which is regardless of the interaction mode. The computed *ϕ*-dependent K-S energy (Fig. 7b) clearly supports this conclusion. Let us extend this argument of the site-dependent MA orientation to the ±B and ±D cases. Considering the structural symmetry rule of {+*A*, −*B*, +*C*, −*D*} for the 1^st^ MA-site, one can readily obtain the reverse conclusion for the ±B and ±D orientations. More specifically, the −B (or −D) orientation is much more stable than the +B (or +D) orientation at the 1^st^ and 3^rd^ sites. On the contrary, +B (or +D) orientation is much more stable than the −B (or −D) orientation at the 2^nd^ and 4^th^ sites of the tetragonal MAPbI_3_.

If the MA dipoles are randomly oriented as in the case of the cubic phase, the number of maximum conceivable orientations of the four MA dipoles in the tetragonal unit cell is given by (2*4)^4^ = 4096 with each orientation represented by characteristic *θ* and *ϕ* angles. Herein, ‘2’ takes into account ± orientations for a fixed *θ*, ‘4’ represents the four possible values of 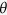 for a fixed *ϕ*, and the power-exponent, 4, takes into account the four distinct MA-sites. Owing to the above symmetry rule of dipole orientations, however, the number of possible orientations of the four MA dipoles in the tetragonal cell can be greatly simplified. To deduce this, suppose that the 1^st^ MA-site is occupied by the MA dipole with the +A orientation under the *α*-interaction mode. Then, −A, +B, −C, and +D orientations are allowed at the 2^nd^ MA-site while +A, −B, +C, and −D orientations are allowed at the 3^rd^ MA-site. Likewise, −A, +B, −C, and +D orientations are allowed at the 4^th^ MA-site. Accordingly, we deduce 64 possible dipole configurations if the 1^st^ MA-site is occupied by the MA dipole with the +A orientation. These 64 dipole configurations are listed in Table 2. Similarly, we have 64 distinct dipole configurations for each occupancy of –B or +C or –D dipole at the 1^st^ MA-site. Thus, we have a total of 256 conceivable dipole configurations in the tetragonal unit cell under the α-hydrogen-bonding interaction mode. Exactly the same number of the dipole configurations is allowed for the *β*-interaction mode but with a different tilting angle, ~+5°. However, the probability of occupying all four MA-sites by the MA dipoles through the *β*-interaction mode is negligible since 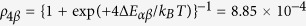, where 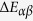 is equal to 45.14 meV. Considering 4096 maximum possible MA configurations, we have achieved a remarkable simplification in the dipole configurations (256/4096 = 1/16) by carefully considering the structural symmetry of the tetragonal MAPbI_3_ cell.

### Evaluation of Individual Hydrogen-bonding Strength

We have shown that the tetragonal MAPbI_3_ perovskite cell under the *α*-interaction mode is much more stable than the same perovskite cell under the *β*-interaction mode with the K-S energy difference of 45.14 meV per MA-site. To quantitatively understand this pronounced mode-dependent structural stability in terms of the strength of the participating hydrogen bonds, we have carefully examined the electron density 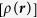 and the corresponding Laplacian of charge density 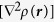 at all the relevant bond critical points (BCPs) by exploiting the so-called ‘quantum theory of atoms in molecules (QTAIM)’^29^. In the QTAIM, the local electronic kinetic-energy density of a given quantum system can be expressed in terms of the first-order density matrix 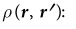:


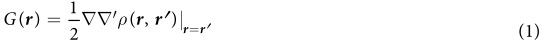


where the local kinetic energy term, 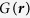, is called “the lagrangian kinetic energy density.” On the basis of QTAIM, Abramov^30^ showed the following expression for 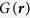 at the BCP, where 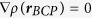:


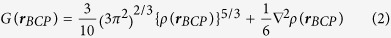


Mata *et al.*^31^ further correlated the hydrogen-bonding energy (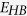) with 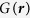 at the BCP using the following relation:


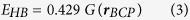


The calculated bonding energy and length, together with the associated topological properties 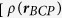 and 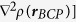, are listed in Table 1 for the 10 relevant H_N_
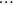
I bonds that are directly involved in the hydrogen-bonding interaction^17^. In addition to this, all ten BCPs (five BCPs for each interaction mode) are marked with small circles in Figure S2 of the Supplementary Information. The well-known criteria of the hydrogen bonding on the basis of QTAIM^32^,^33^ are (i) 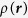 between 0.002 and 0.034 *a.u.* (atomic unit) and (ii) 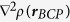 between +0.024 and +0.139 *a.u.* at the BCP, where 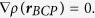 A certain degree of the flexibility is effectively allowed in the hydrogen-bonding criteria^33^, especially in the range of 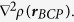 Thus, all the H_N_
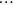
I bonds listed in Table 1 practically satisfy the criteria of the hydrogen bonding. Among 10 different hydrogen bonds, three are prominent in their 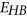 values and bonding lengths (<2.70Å). Previously, they are named ‘the dominant hydrogen bonds’ (Subsection “Two Distinct Modes of Hydrogen-bonding Interaction”) and are: H_N_(1)
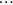
I_A_(1) and H_N_(2)
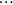
I_A_(2) in the *α*-interaction mode and H_N_(3)
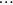
I_E_(1) in the *β*-interaction mode.

According to the computed results shown in Table 1, the net difference in the hydrogen-bonding energy 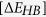 between the two interaction modes is 43.87 meV (=381.06–337.19) per formula cell. This clearly supports the previously made conclusion that the tetragonal MAPbI_3_ perovskite cell under the *α*-interaction mode is much more stable than the same perovskite cell under the *β*-interaction mode (Subsection “Two Distinct Modes of Hydrogen-bonding Interaction”). Moreover, the estimated bonding-energy difference by the QTAIM (43.87 meV) nearly coincides with the previously calculated K-S energy difference between the two interaction modes (45.14 meV). As indicated in Eqs. (2) and (3), the computed 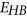 value by the QTAIM depends on 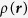 at the BCP. In the DFT, 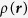 uniquely determines the external potential^34^, thus, the ground-state K-S energy that comprises all the interaction terms including the Hartree energy, the external interaction energy between the nucleus and electrons, and the exchange-correlation energy. Thus, the computed value of 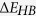 (43.87 meV) by applying the QTAIM can be viewed as the overall K-S energy difference between the two interaction modes (45.14 meV), rather than being interpreted as the difference in the pure hydrogen-bonding interaction energy between the two interaction modes.

## Conclusion

On the basis of symmetry argument and DFT calculations, we have made the following conclusions on the tetragonal MAPbI_3_ perovskite cell: **(i)** There exist two distinct types of the hydrogen-bonding interaction between the MA^+^-ion and the iodide ions in the PbI_6_-octahedron network. We named them *α*- and *β*-interaction modes. **(ii)** The computed K-S energy difference between these two interaction modes is 45.14 meV per MA-site with the *α*-interaction mode being responsible for the stable hydrogen-bonding network. **(iii)** Based on the individual bonding-energy calculations by exploiting the QTAIM, we have shown that five distinct hydrogen bonds are effective in the tetragonal MAPbI_3_ under the stable *α*-interaction mode. The net difference in the total hydrogen-bonding energy between these two interaction modes is 43.87 meV per MA-site, which nearly coincides with the K-S energy difference of 45.14 meV. **(iv)** We have further made a remarkable simplification in the MA-dipole configurations by imposing the structural symmetry rule and the tilting-angle-dependent K-S energy to the tetragonal MAPbI_3_ cell.

## Methods

We have performed *ab initio* density functional theory (DFT) calculations on the basis of the Perdew-Burke-Enzerhof generalized gradient approximation (PBE-GGA)^35^,^36^ implemented with projector augmented-wave (PAW) pseudopotential^37^ using the Vienna *ab initio* Simulation Package (VASP)^38^,^39^. To assess the effect of the van der Waals (vdW) interaction on the structure relaxation, we have performed all *ab initio* calculations using the internal parameters of the Grimme’s DFT-D2 vdW as implemented in VASP^40^. Most of the DFT calculations were performed by adopting (i) a 4x4×3 Monkhorst-Pack (M-P) ***k***-point mesh^41^ centered at the Γ-point and (ii) a 500-eV plane-wave cutoff energy. In the band-structure calculations, however, we have initially adopted a 3x3×2 M-P ***k***-point mesh to obtain a relaxed structure and subsequently used a 9x9×6 ***k***-point mesh to accurately assess the ***k***-point-dependent Kohn-Sham energy. All the structural relaxations were performed with a Gaussian broadening of 0.05 eV. The ions were relaxed until the Hellmann-Feynmann forces on them were less than 0.01 eV•Å^−1^. The topological analysis of electronic density contours was performed by suitably exploiting the AIM-UC program^42^.

## Additional Information

**How to cite this article**: Lee, J. H. *et al.* The nature of hydrogen-bonding interaction in the prototypic hybrid halide perovskite, tetragonal CH_3_NH_3_PbI_3_. *Sci. Rep.* **6**, 21687; doi: 10.1038/srep21687 (2016).

## Supplementary Material

Supplementary Information

## Figures and Tables

**Table 1:** The calculated electronic topological properties, together with the bonding energy, length and angle, for the 10 relevant H_N_
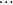
I bonds that are directly involved in the two distinct modes of the hydrogen-bonding interaction.

BCP	*ρ* (*a.u.*)	∇^2^*ρ* (*a.u.*)	E_HB_(meV)	Bonding length (Å)	Bonding angle (°)
*α*-mode					
H_N_(1) 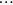 I_A_(1)	0.01747	0.03072	99.20	2.64	171.1
H_N_(2) 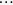 I_A_(2)	0.01706	0.03052	97.28	2.65	165.9
H_N_(3) 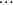 I_E_(2)	0.00882	0.02410	59.61	3.04	119.9
H_N_(3) 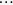 I_E_(3)	0.00994	0.02437	62.95	2.96	126.7
H_N_(3) 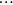 I_E_(4)	0.00958	0.02419	62.02	2.98	122.3
	Total E_HB_ of *α*-mode		**381.06**(meV)		
*β*-mode					
H_N_(1) 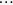 I_A_(1)	0.00913	0.02397	59.92	2.99	132.1
H_N_(1) 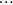 I_E_(4)	0.01070	0.02408	64.27	2.91	133.0
H_N_(2) 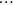 I_A_(2)	0.01007	0.02408	62.58	2.95	133.3
H_N_(2) 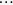 I_E_(3)	0.01038	0.02401	63.12	2.93	132.1
H_N_(3) 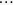 I_E_(1)	0.01603	0.02731	87.30	2.68	175.9
	Total E_HB_ of *β*-mode		**337.19**(meV)		

**Table 2:** 64 symmetry-allowed dipole configurations for the occupation of four distinct MA-dipole sites in the tetragonal MAPbI_3_ unit cell when the 1^st^ MA-site is occupied by the MA dipole with the +A orientation.

1st	2nd	3rd	4th	1st	2nd	3rd	4th	1st	2nd	3rd	4th	1st	2nd	3rd	4th
+A	−A	+A	−A	+A	+B	+A	−A	+A	−C	+A	−A	+A	+D	+A	−A
+A	−A	+A	+B	+A	+B	+A	+B	+A	−C	+A	+B	+A	+D	+A	+B
+A	−A	+A	−C	+A	+B	+A	−C	+A	−C	+A	−C	+A	+D	+A	−C
+A	−A	+A	+D	+A	+B	+A	+D	+A	−C	+A	+D	+A	+D	+A	+D
+A	−A	−B	−A	+A	+B	−B	−A	+A	−C	−B	−A	+A	+D	−B	−A
+A	−A	−B	+B	+A	+B	−B	+B	+A	−C	−B	+B	+A	+D	−B	+B
+A	−A	−B	−C	+A	+B	−B	−C	+A	−C	−B	−C	+A	+D	−B	−C
+A	−A	−B	+D	+A	+B	−B	+D	+A	−C	−B	+D	+A	+D	−B	+D
+A	−A	+C	−A	+A	+B	+C	−A	+A	−C	+C	−A	+A	+D	+C	−A
+A	−A	+C	+B	+A	+B	+C	+B	+A	−C	+C	+B	+A	+D	+C	+B
+A	−A	+C	−C	+A	+B	+C	−C	+A	−C	+C	−C	+A	+D	+C	−C
+A	−A	+C	+D	+A	+B	+C	+D	+A	−C	+C	+D	+A	+D	+C	+D
+A	−A	−D	−A	+A	+B	−D	−A	+A	−C	−D	−A	+A	+D	−D	−A
+A	−A	−D	+B	+A	+B	−D	+B	+A	−C	−D	+B	+A	+D	−D	+B
+A	−A	−D	−C	+A	+B	−D	−C	+A	−C	−D	−C	+A	+D	−D	−C
+A	−A	−D	+D	+A	+B	−D	+D	+A	−C	−D	+D	+A	+D	−D	+D
