# Supplementary material for: The nature of hydrogen-bonding interaction in the prototypic hybrid halide perovskite, tetragonal CH3NH3PbI3
Source: Sci Rep. 2016 Feb 19;6:21687. doi: 10.1038/srep21687 (PMC4759593; doi:10.1038/srep21687)
Supplement: Supplementary Information [file srep21687-s1.pdf]

Supplementary Information

The nature of hydrogen-bonding interaction in the prototypic hybrid halide perovskite, tetragonal $CH_3NH_3PbI_3$

June Ho Lee,[†] Jung-Hoon Lee,[†] Eui-Hyun Kong,[‡] and Hyun Myung Jang*[†]

[†] Department of Materials Science and Engineering, and Division of Advanced Materials Science (AMS), Pohang University of Science and Technology (POSTECH), Pohang 790-784, Republic of Korea.

[‡] Korea Atomic Energy Research Institute (KAERI), Yuseong-Gu, Daejeon 305-353, Republic of Korea.

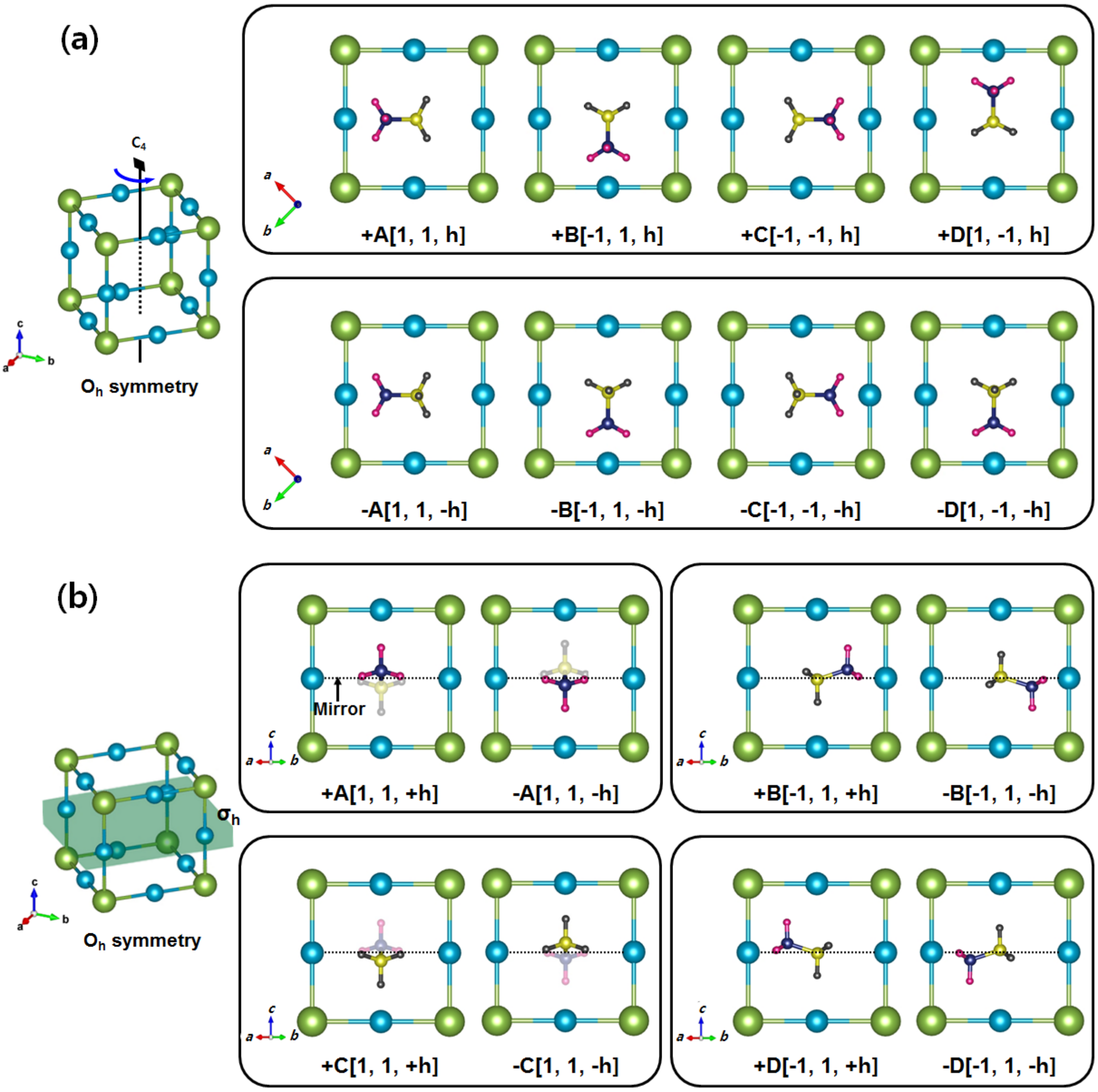

Figure S1. Graphical illustration of eight MA-dipole orientations having the same chemical environment in the cubic $MAPbI_3$ cell. **(a)** Two distinct sets of the MA-dipole orientations, $\{+A, +B, +C, +D\}$ and $\{-A, -B, -C, -D\}$, as obtained by the principal C_4 rotation operation. **(b)** Four distinct types of the MA-dipole orientations, as obtained by the reflection through the mirror plane (σ_h) perpendicular to the C_4 rotation axis.

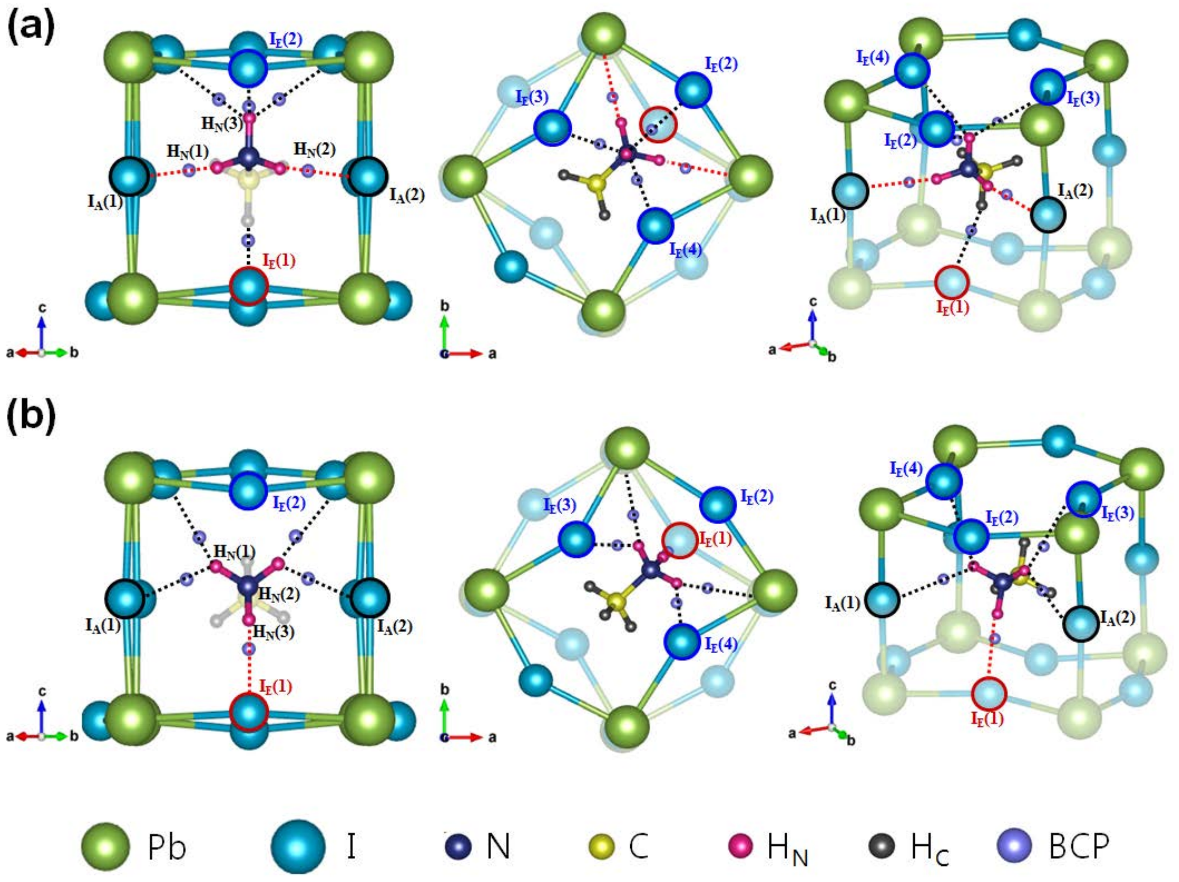

Figure S2. Illustration of the two distinct modes of the hydrogen-bonding interaction between the MA^{+}-ion and the surrounding PbI_6-octahedron cages. **(a)** α-interaction mode viewed along [110] (left), viewed along [001] (center), and viewed from an arbitrary axis (right). **(b)** β-interaction mode viewed along [110] (left), viewed along [001] (center), and viewed from an arbitrary axis (right). The ten relevant H_N···I bonds directly involved in the hydrogen-bonding interaction (five per each mode) are denoted by dotted lines. In addition to this, all ten BCPs (five BCPs for each mode) are marked with small circles.
